# Plank Side Walks

**Published:** 1882-06-20

**Authors:** R. L. Hixton

**Affiliations:** President Board of Health, Prescott, Ark.


### *PLANK SIDE WALKS—THEIR EFFECT UPON HEALTH.*

Dr. R. L. Hinton, writes:

Mr. EDITOR.—Will you allow me space to ask for information in reference to plank side-walks, as regards their effect upon the health, when extensively used. Information from any of my professional brethren who have had sufficient observation to give anything like a positive and definite reply, will be thankfully received.

Respectfully, etc.

R. L. HINTON,

President Board of Health, Prescott, Ark.

[Will be pleased to have a response to the above inquiry, and if Dr. H. himself has any special view on the subject, we hope he will give them to the Profession through our Journal—ED.]

